# Pulmonary embolism therapies and outcomes: Hospital registries, industry sponsored trials, and the impact of the PERT consortium

**DOI:** 10.1016/j.jvsv.2024.101824

**Published:** 2024-04-15

**Authors:** Patrick E. Muck

**Affiliations:** Department of Vascular Surgery, TriHealth - Good Samaritan Hospital Cincinnati, Cincinnati, Ohio, USA

The authors did an outstanding job with their registry examining patients with pulmonary embolism (PE) undergoing reperfusion therapy through the Pulmonary Embolism Response Team (PERT).^1^ PE Interventions, both catheter-directed thrombolysis (CDT) and large-bore mechanical/aspiration thrombectomy are not without risks. These include intracranial hemorrhage, retroperitoneal bleeding, pulmonary artery perforation, and cardiac arrhythmias. Cardiac arrest can occur with the passage of large-bore devices across the right heart. The risks and benefits of these reperfusion therapies as well as the long-term benefits can be studied through single-center hospital or system registries. In the present study, Bashir et al.^2^ studied CDT with a specialized catheter finding a significant reduction in total and subtotal occlusion of segmental and proximal pulmonary arteries. Semaan et al.^3^ concluded that CDT plus anticoagulation was associated with improved long-term survival compared with anticoagulation alone for PE patients.

Multicenter Industry sponsored trials are typically needed for Food and Drug Administration (FDA) approval of new devices. Multiple CDT and thrombectomy trials have shown reperfusion therapies to be safe and efficacous.^4^^,^^5^ However, these trials have strict inclusion and exclusion criteria and rarely have follow-up beyond 30 days. Industry-sponsored trials are crucial for the advancement of technology, but perhaps are not always applicable for real-world patients. The more ill, complex patients that physicians see daily are excluded from trials, eliminating the true known risk profile of reperfusion devices. Ultimately, the goal for industry sponsored trials is FDA device approval. Name the last trial your institution was involved in that did not lead to FDA device approval.

Undoubtedly, a randomized controlled trial would answer many questions about the risks and benefits of reperfusion therapies. In the absence of a randomized controlled trial, a multicenter multidisciplinary registry can help identify the short- and long-term results of reperfusion devices in real-world patients. Here enters the PERT Consortium and its PERT Quality Assurance Database. The first PERT was created in 2012 at Massachusetts General Hospital and the PERT Consortium followed in 2014. The Consortium's mission is to increase awareness of treatment options available to patients with PE, decrease the worldwide incidence of PE and further scientific discovery in PE research (https://pertconsortium.org/). PERT programs can be implemented, with similar structures, at small and large, community and academic medical centers. The PERT Quality Assurance Database is a multicenter registry of patient-level data from patients admitted to participating centers managed by their PERTs. The PERT Consortium Quality Assurance Database works very much like the Vascular Quality Initiative. Participating centers receive quarterly comparative reports on processes and outcomes. The PERT Consortium database committee has regular calls for research projects that are reviewed, much as is done with Vascular Quality Initiative participation. The PERT Consortium has published extensively. It is an excellent example of a team-centered approach to research and development of management guidelines.^6^, ^7^, ^8^, ^9^

Outside of randomized controlled trials, registries and large databases allow for real-world data analysis without the strict criteria of a clinical trial. The PERT Consortium is a refreshing example of the utility of multicenter, multidiscipline collaboration. ST-segment elevation myocardial infarction and stroke teams are examples of a collaborative approach and are clearly the standard of care for those patient populations. Dr Charles Ross, a vascular surgeon served on the original board of directors of the PERT Consortium. He notes that the consortium is very accepting of vascular surgeon participation and offers abundant opportunities for professional growth. I encourage vascular surgeons to join or help in the formation of PERTs in their institutions and to join the PERT Consortium. Collaboration will help to establish benchmarks for treatment with reperfusion therapies for patient with PE. I agree with the authors, this submission suggests the possibility of expanding reperfusion therapies beyond current recommendations. Hopefully, future randomized control trials can evaluate these technologies, until then we have the PERT Consortium leading the way.

*The opinions or views expressed in this commentary are those of the authors and do not necessarily reflect the opinions or recommendations of the Journal of Vascular Surgery: Venous and Lymphatic Disorders or the Society for Vascular Surgery.*

## Disclosures

None.
